# Predicting protein-protein interface residues using local surface structural similarity

**DOI:** 10.1186/1471-2105-13-41

**Published:** 2012-03-18

**Authors:** Rafael A Jordan, Yasser EL-Manzalawy, Drena Dobbs, Vasant Honavar

**Affiliations:** 1 Department of Computer Science, Iowa State University, Ames, IA 50011, USA; 2 Department of Genetics, Development and Cell Biology, Iowa State University, Ames, IA 50011, USA; 3 Bioinformatics and Computational Biology Program, Iowa State University, Ames, IA 50011, USA; 4 Department of Systems and Computer Engineering, Pontificia Universidad Javeriana, Cali, Colombia; 5 Department of Systems and Computer Engineering, Al-Azhar University, Cairo, Egypt

## Abstract

**Background:**

Identification of the residues in protein-protein interaction sites has a significant impact in problems such as drug discovery. Motivated by the observation that the set of interface residues of a protein tend to be conserved even among remote structural homologs, we introduce *PrISE*, a family of local structural similarity-based computational methods for predicting protein-protein interface residues.

**Results:**

We present a novel representation of the surface residues of a protein in the form of structural elements. Each structural element consists of a central residue and its surface neighbors. The *PrISE *family of interface prediction methods uses a representation of structural elements that captures the atomic composition and accessible surface area of the residues that make up each structural element. Each of the members of the *PrISE *methods identifies for each structural element in the query protein, a collection of *similar *structural elements in its repository of structural elements and weights them according to their similarity with the structural element of the query protein. *PrISE_L _*relies on the similarity between structural elements (i.e. local structural similarity). *PrISE_G _*relies on the similarity between protein surfaces (i.e. general structural similarity). *PrISE_C_*, combines local structural similarity and general structural similarity to predict interface residues. These predictors label the central residue of a structural element in a query protein as an interface residue if a weighted majority of the structural elements that are similar to it are interface residues, and as a non-interface residue otherwise. The results of our experiments using three representative benchmark datasets show that the *PrISE_C _*outperforms *PrISE_L _*and *PrISE_G_*; and that *PrISE_C _*is highly competitive with state-of-the-art structure-based methods for predicting protein-protein interface residues. Our comparison of *PrISE_C _*with *PredUs*, a recently developed method for predicting interface residues of a query protein based on the known interface residues of its (global) structural homologs, shows that performance superior or comparable to that of *PredUs *can be obtained using only local surface structural similarity. *PrISE_C _*is available as a Web server at http://prise.cs.iastate.edu/

**Conclusions:**

Local surface structural similarity based methods offer a simple, efficient, and effective approach to predict protein-protein interface residues.

## Background

Protein-protein interactions play a central role in many cellular functions. In the past decade, significant efforts have been devoted to characterization as well as discovery of these interactions both in silico and in vivo [1-5]. Of particular interest is the identification of the amino acid residues that participate in protein-protein interactions because of its importance in elucidation of mechanisms that underlay biological function and rational drug design (among other applications) [6]. However, experimental determination of interface residues is expensive, labor intensive, and time consuming [7]. Hence, there is an urgent need for computational methods for reliably identifying from the sequence or structure of a query protein, the subset of residues that are likely to be involved in the interaction of that protein with one or more other proteins.

Several methods for predicting protein-protein interface residues have been proposed in the literature (see the reviews in [8-10]). A variety of features of the target residue (and often its sequence or structural neighbors) have been explored [11,12] in combination with machine learning techniques [13-23] or scoring functions [24-29] to construct predictors of interface residues. Of particular interest are recent methods for protein interface prediction based on the structural similarity between a query protein and proteins with known structure. These methods are motivated by observations that suggest that interaction sites tend to be conserved among structurally similar proteins [30-34]. As the number of experimentally determined complexes in the Protein Data Bank (PDB) [35] increases, the likelihood of success of such an approach to interface prediction can be expected to increase as well. Hence, there is growing interest in structural similarity based approaches to protein-protein interface prediction. For example, Konc and Janežič [36] and Carl et al. [37] developed a method that utilizes a graph based representation of protein surfaces to predict interface residues that exploits the higher degree of conservation of topological and physico-chemical features among interaction sites as compared to non-interaction sites of proteins. Zhang et al. [38] introduced *PredUs*, a new method that predicts interaction sites using counts of interface residues derived from alignments between the structure of a query protein and the structures of a set of proteins that are structurally similar to the query protein. More recently, *PredUs *has been updated [39] to incorporate a support vector machine that uses accessible surface area of regions on the protein surface and the counts of interface residues derived from the structural alignments to predict interface residues.

A potential limitation of structural similarity based interface prediction methods is that they are effective only to the extent that a set of proteins (with experimentally determined interface residues) that are structurally similar to the query protein can be reliably identified. In light of evidence that the degree of conservation of interfaces tends to be substantially higher than that of non-interfaces [30] and hence that of whole protein structures, there is increasing interest in methods for predicting interface residues based on experimentally determined interface residues in proteins that are locally (as opposed to globally) similar in structure to the query protein [40,41].

Against this background, we introduce *PrISE *(Predictor of Interface Residues using Structural Elements), a novel family of predictors of protein-protein interface residues based on local structural similarity. The *PrISE *family of interface prediction methods utilizes a repository of structural elements constructed from a dataset of proteins that are part of experimentally determined protein complexes retrieved from the PDB. A structural element is defined as a protein surface residue surrounded by its neighbors on the protein surface. The *PrISE *methods utilize a novel representation of each structural element that captures the distribution of the constituent atoms and the solvent accessible surface areas of residues (calculated from the individual proteins). The prediction of protein-protein interface residues using any of the *PrISE *methods is based on the identification of a collection of structural elements in the repository that are *similar *to the structural elements of a query protein. The *PrISE *predictors label the central residue of each structural element in the query protein as an interface residue if a weighted majority of the similar structural elements are interface residues and as a non-interface residue otherwise. *PrISE_L _*relies on the similarity between structural elements to assign the weights to each query structural element whereas *PrISE_G _*relies on the similarity between protein surfaces in terms of structural elements. *PrISE_C _*combines the local and global approaches of *PrISE_L _*and *PrISE_G_*. We assessed the performance of the *PrISE *family of predictors using several benchmark datasets. The results of experiments show that *PrISE_C _*outperforms *PrISE_L _*and *PrISE_G_*. The three *PrISE *family of predictors outperform two other local structural similarity based interface residue predictors [37,41]. *PrISE_C _*also outperforms methods that use diverse structural, evolutionary, and physico-chemical properties to perform prediction of interface residues using machine learning and scoring functions, even in the absence of proteins with similar structure. The performance of *PrISE_C _*is superior or comparable to that of *PredUs *[38,39], a novel method that predict interface residues using the known interface residues on proteins with similar structure to a query protein. Unlike *PredUs*, that require the existence of structural homologs to perform predictions, *PrISE_C _*is able to generate prediction for all the proteins with known structure.

## Methods

### Structural elements and their representation

*A structural element *is defined by an amino acid residue on the protein surface (referred to as *a surface residue*) and its neighboring surface residues. Thus, the number of structural elements in a protein equals the number of its surface residues. An amino acid residue is considered a *surface residue *if its accessible surface area in the monomer is greater than zero. Two residues are considered neighbors if the distance between the Van der Waals surface of an *atom *of one residue and the Van der Waals surface of an *atom *of the other residue is ≤ 1.5 Å. Accessible surface areas were computed using Naccess [42].

A structural element is represented using four features: (i) The name of the central residue of the structural element; (ii) the accessible surface area of the central residue of the structural element; (iii) the accessible surface area of the structural element (computed as the addition of the accessible surface areas of its residues); and (iv) a histogram of atom nomenclatures representing the atomic composition of the surface of the structural element. A *histogram of atom nomenclatures *contains the count of the number of atoms on the surface of the structural element for each atom nomenclature (e.g. number of *α*-carbons, number of *β*-carbons, etc.). There are 36 atom nomenclatures (a list is presented in section one of the Additional File 1), hence, a histogram of atom nomenclatures has 36 bins. An atom is considered to be in the surface of a protein if its accessible surface area is > 0 Å^2^. The four features that represent a structural element are used to define a similarity measure between structural elements that consider structural and physico-chemical properties. The rationale behind this representation is that structural elements with similar accessible surface areas and centered on identical residues with similar surface areas have similar structure. In addition, two structural elements with similar atomic composition of the surface of the structural element (represented by the histogram of atom nomenclatures) have similar physico-chemical properties.

### Distance between histogram of atom nomenclatures

The distance between the histograms of atom nomenclatures of two structural elements provides a measure of their physico-chemical similarity. The distance between two histograms of atom nomenclatures *x *and *y *is computed using the city block metric: $\sum_{i=1}^{36}\left|x_{i}-y_{i}\right|$, where *x_i _*and *y_i _*denote the number of atoms (corresponding to the *i^th ^*nomenclature in the histograms) on the surface of the two structural elements (e.g. number of *α*-carbons exposed to the solvent)^a^.

### Repository of structural elements

*A repository of structural elements *stores all the structural elements extracted from a set of proteins. To perform different experiments, we built two repositories from two different sets of proteins. The first, called the *ProtInDB repository*, was built from the biological assemblies stored in *ProtInDB *[43], a database of protein-protein interface residues, which in turn was derived from protein complexes in PDB [35]. This repository is composed of 21,289,060 structural elements extracted from 88,593 interacting chains (as of February 21, 2011). The second repository, called the *ProtInDB *⋂ *PQS repository*, is composed of the structural elements extracted from proteins that are common to both *ProtInDB *and the Protein Quaternary Structure database (*PQS*) [44]. This repository contains 13,396,420 structural elements extracted from 55,974 interacting chains in 21,786 protein complexes. A protein chain is considered an *interacting chain *if it contains at least five *contact *amino acid residues. An amino acid residue in a protein chain is considered a contact amino acid if the Van der Waals surface of at least one of its heavy atoms is no further than at most 0.5 Å from the Van der Waals surface of some heavy atom(s) of an amino acid residue belonging to another chain.

### Retrieving similar structural elements

The prediction of interface residues in a query protein is based on the existence of similar structural elements for each structural element in the protein. The process of retrieval similar structural elements from a repository of structural elements should satisfy two requirements: It should be efficient and it should retrieve similar structural elements for every structural element in the query protein. These requirements are satisfied using four constraints that every structural element *q_s _*retrieved from the repository and associated with a query structural element *q_r _*should comply: (i) *q_r _*and *q_s _*must not be from the same protein complex; (ii) the central residues *r *and *s *of the structural elements *q_r _*and *q_s _*respectively, must be identical; (iii) the difference between the accessible surface areas of *r *and *s *should be ≤ 5% of the maximum accessible surface area of residues identical to *r*; and (iv) the differences between the accessible surface areas of *q_r _*and *q_s _*must be ≤ 15% of the maximum estimated accessible surface area of any structural element centered on a residue identical to *r*. These constraints were experimentally determined, as explained in the Additional File 1.

### PrISE algorithm

**Figure 1:**
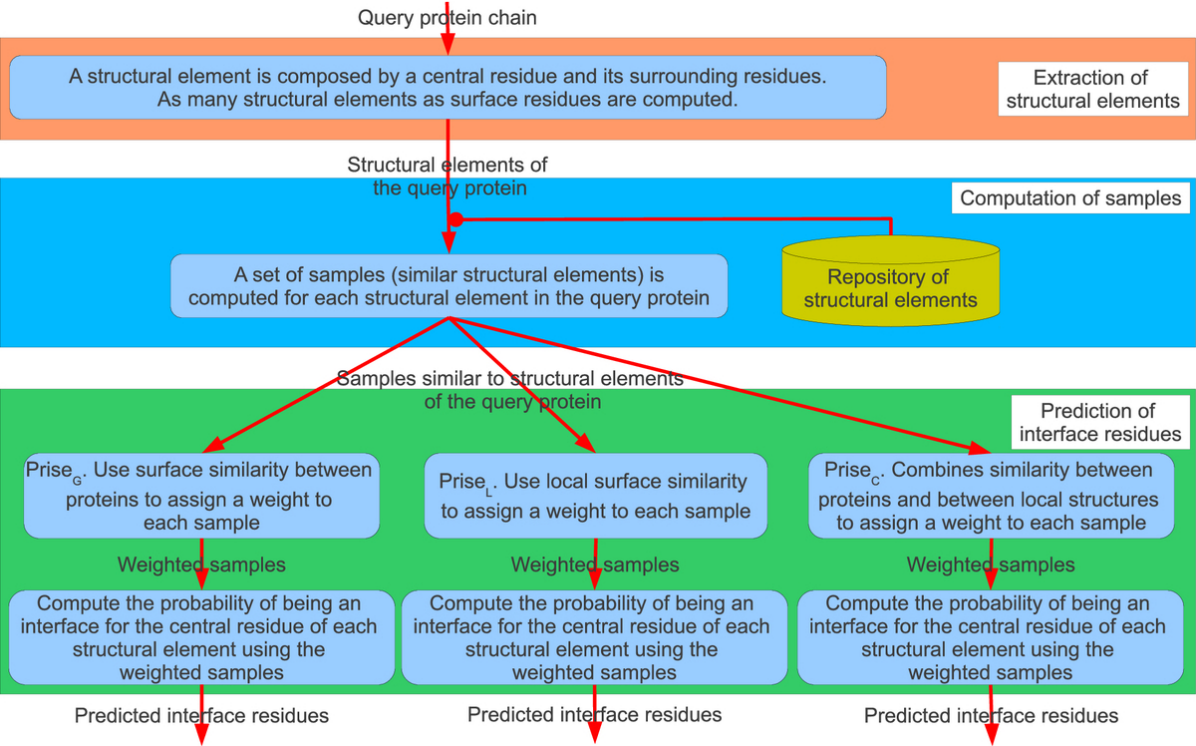
**Prediction of interface residues using surface structural similarity**.

The *PrISE *algorithm is summarized in Figure 1. First, a query protein structure is decomposed into a collection of structural elements. For each structural element in the query protein, *PrISE *retrieves a collection of similar structural elements (referred as samples) from the repository of structural elements. *PrISE *uses the collection of retrieved samples and information derived from their associated proteins to predict whether the central residue of each structural element is an interface residue. The information derived from the associated proteins can be incorporated into our proposed method using three different approaches (Equations 1-3) that result in three variants of the *PrISE *algorithm for predicting protein interface residues. The first method, *PrISE_L_*, uses similarity between structural elements (i.e. local structural similarity). The second method, *PrISE_G_*, utilizes a measure of similarity between protein surfaces (i.e. general structural similarity). The last method, *PrISE_C_*, combines local and general structural similarity. A detailed description of these approaches as well as the rationales behind them are provided next.

Let *S *be a repository of structural elements (where each element is indexed by the protein from which the structural element is derived and the surface residue that it represents). Let *Q *be a query protein. Let *S(Q) *be the collection of structural elements of *Q *(recall that there are as many structural elements in *S(Q) *as there are surface residues in *Q*). To predict whether the central residue *r*(*q*) of a structural element *q *∈ *S*(*Q*) is an interface residue, a collection *S_q _*of structural elements that are most similar to *q *is retrieved from the repository *S *based on the distance between the histogram of atom nomenclatures *q *and that of each element in *S^b^*. In the event of a tie, the sample with the lowest difference in accessible surface area between its central residue and residue *r(q) *is chosen.

For each structural element *s *in *S*, let π(s) denote the protein from which *s *was extracted. Given a protein *P *and an arbitrary collection *R *of structural elements, we define the *contribution, cont(P, R)*, as the number of structural elements in *R *that are associated with the protein *P*. For each *q *∈ *S *(*Q*), the collection of structural elements of protein *Q*, and for each structural element *s *∈ *S*_*q*_, we define the *weights w_G_(s, q), w_L_(s, q) *and *w_C_(s, q) *(used by *PrISE_G_, PrISE_L_*, and *PrISE_C _*respectively) as follows:

(1) $$w_{G}\left(s,q\right)=cont\left(\pi \left(s\right),z_{Q}\right)$$

where $z_{Q}=\underset{q\in S\left(Q\right)}{\cup }S_{q}$. Intuitively, the more similar the query protein *Q *containing the structural element *q *is to the protein from which the structural element *s *was derived, the greater the influence of s to the prediction on *q*.

Given a structural element *q *∈ *S*(*Q*), let *Re(q) *be the set of surface residues of *Q *that belong to *q*. Let *N*(*q*) be the set of structural elements associated with residues in *Re(q)*. Let $N_{q}=\underset{n\in N\left(q\right)}{⋃}S_{n}$ (where *S_n_*, the collection of structural elements that are most similar to *n*, is retrieved from the repository *S *of structural elements), we define the weight for *PrISE_L _*as:

(2) $$w_{L}(s,q)=cont(\pi (s),N_{q}))$$

Intuitively, the more similar the local surface patch of the structural element *q *is to a local surface patch of the protein from which the structural element *s *was derived, the greater the influence of s to the prediction on *q*.

For *PrISE_C_*,

(3) $$w_{c}\left(s,q\right)=w_{G}\left(s,q\right)\times w_{L}\left(s,q\right)$$

Let *S*_+_(*q*) = {s ∈ *S_q_*|*r*(*s*) is an ineterface residue} and *S*_-_(*q*) = {s ∈ *S*_*q*_|*r*(*s*) is a non-interface residue}. Thus, *PrISE_C _*combines the predictions of *PrISE_L _*and *PrISE_G_*. Because *PrISE_L _*and *PrISE_G _*weight each sample based on different criteria, this allows *PrISE_C _*potentially to outperform each of them by taking advantage of complementary methods.

In the case of *PrISE_G_*, the weight of positive samples associated with structural element *q *is defined as:

$${W_{G}}_{+}\left(q\right)=\underset{s\in S_{+}\left(q\right)}{\sum }w_{G}\left(s,q\right)$$

Similarly, the weight of negative samples associated with structural element *q *is defined as:

$${W_{G}}_{-}\left(q\right)=\underset{s\in S_{-}\left(q\right)}{\sum }w_{G}\left(s,q\right)$$

Finally, classification is performed by selecting a threshold ^*c *^on the probability that indicates whether the central residue *r*(*q*) of the structural element *q *is likely to be an interface residue:

$$pro{b_{G}}_{+}\left(r\left(q\right)\right)=\frac{{W_{G}}_{+}\left(q\right)}{{W_{G}}_{+}\left(q\right)+{W_{G}}_{-}\left(q\right)}$$

In the case of *PrISE_L_*, and *PrISE_C_*, the corresponding quantities *W_L_*_+_(*q*), *W_L_*_-_(*q*), and *prob_L_*_+_(*r*(*q*)) and *W_C_*_+_(*q*), *W_C_*-(*q*), and *prob_C_*_+_(*r*(*q*)) are defined in terms of the corresponding weights *w_L _*and *w_C _*(respectively).

### Datasets

Four datasets were used to assess the performance of the *PrISE *family of interface predictors. The first dataset, DS24Carl [37], is composed of 24 chains: 16 extracted from transient complexes and eight extracted from complexes of different types. In this dataset, a residue is defined as an *interface residue *if the distance of the Van der Waals surface of any of its heavy atoms to a Van der Waals surface in any heavy *atom *of a different chain is ≤ 3 Å. The other three datasets were defined in [38] from complexes used to evaluate protein docking software. DS188 is composed of 188 proteins chains derived from the Docking Benchmark 3.0 [45] sharing at most 40% sequence identity and containing 39,799 residues and 7,419 interacting residues. The other two datasets, DS56bound and DS56unbound, are composed by 56 protein chains derived from bound and unbound structures from the first 27 targets in CAPRI [46]. DS56bound and DS56unbound have a total of 12,123 and 12,173 residues, and 2,154 and 2,112 interacting residues respectively. For these three datasets, interface residues are defined as amino acids on two different protein chains with at least a pair of heavy atoms separated by at most 5 Å. These interfaces were computed from complexes extracted from PQS by the authors of [38].

### Performance evaluation

The reliability of a prediction may be evaluated using different performance measures [47]. We focused our evaluation on the following measures:

$$precision=\frac{TP}{TP+FP}$$

$$recall=\frac{TP}{TP+FN}$$

where *TP *refers to interface residues correctly predicted, *FP *to non-interface residues predicted as interfaces, and *FN *to interface residues predicted as non-interfaces. *Precision *evaluates the quality of the prediction in reference to the set of predicted interface residues, whereas *recall *measures the quality of the prediction with respect to the set of actual interface residues. When possible, the performance of different classifiers is evaluated by comparison of the precision-recall curve of each classifier. These curves are generated by computing precision and recall using different threshold values on the probability of each residue to be part of the interface. Therefore, these curves provide a more comprehensive evaluation than a pair of precision and a recall values.

For sake of completeness, we computed the following measures:

$$F1=\frac{2\times precision\times recall}{precision+recall}$$

$$Accuracy=\frac{TP+TN}{N}$$

$$CC=\frac{\left(TP\times TN\right)-\left(FP\times FN\right)}{\sqrt{\begin{array}{c} \left(TP+FN\right)\times \left(TP+FP\right)\times \\ \times \left(TN+FP\right)\times \left(TN+FN\right) \end{array}}}$$

The F1 score computes the harmonic mean between precision and recall. Accuracy measures how well interface and non-interface residues are correctly predicted. CC refers to the Matthews correlation coefficient. In addition, we use the area under the receiver operating characteristic (AUC ROC). This measure computes the area under the curve generated by computing the sensitivity and the false positive rate using different thresholds on the probabilities that indicates whether a residue belongs to the interface.

## Results and discussion

We compared the *PrISE *family of algorithms using the DS188, DS24Carl, DS56bound and DS56unbound datasets. We also assessed the extent to which the quality of predictions is impacted by the presence of structural elements derived from homologs of the query protein in the repository of structural elements used to make the predictions. In addition, the performance of *PrISE_C _*was assessed against the performance of several classifiers based on machine learning methods, scoring functions, and local and global structural similarity on different datasets.

### Comparison of PrISE_L_, PrISE_G _and PrISE_C_

**Figure 2:**
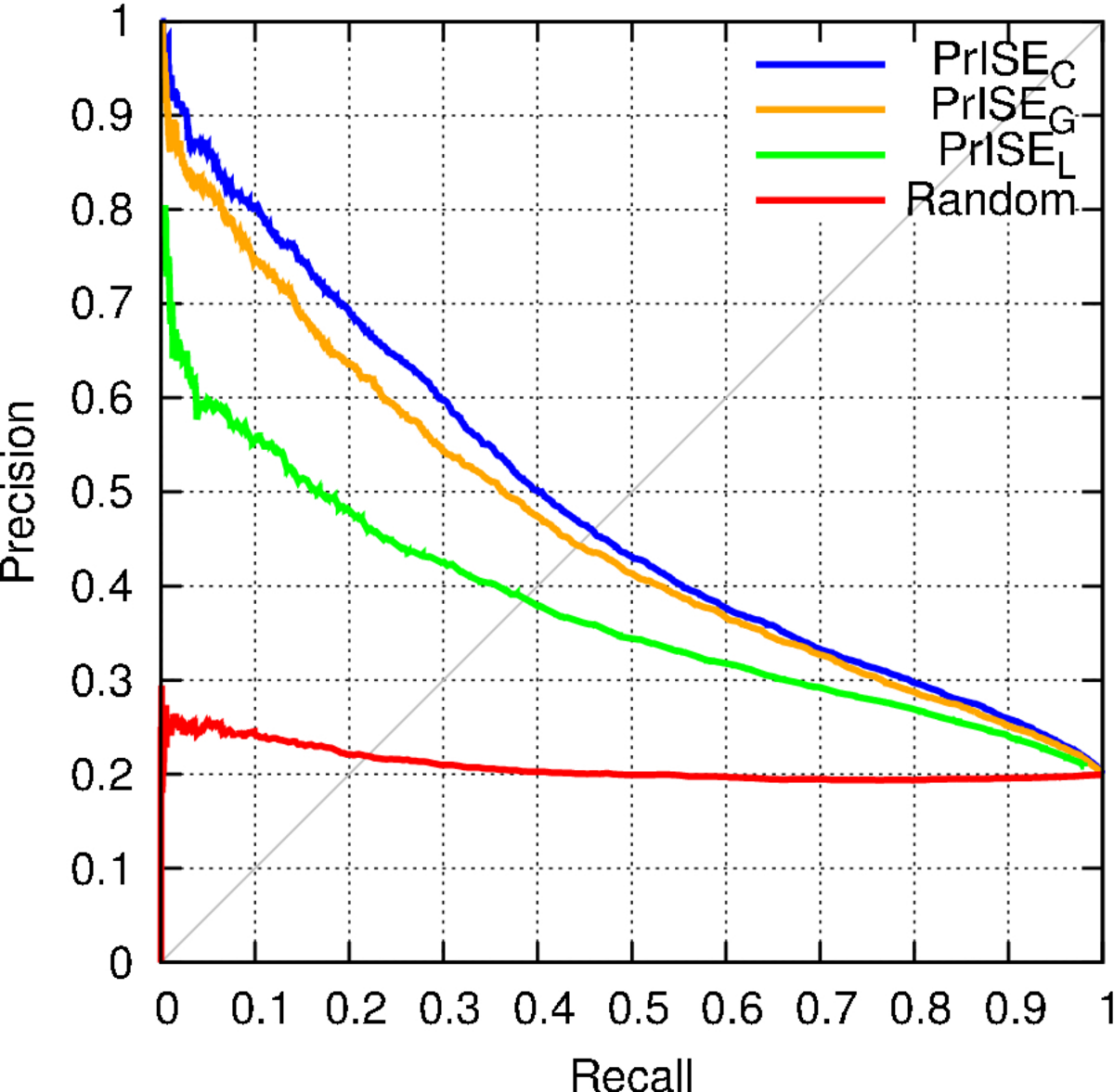
**Comparative performances of PrISE_L_, PrISE_G_, PrISE_C_, and randomly generated predictions on the DS188 dataset**.

Recall that *PrISE_L _*relies on the similarity between structural elements (i.e. local structural similarity), *PrISE_G _*relies on the similarity between protein surfaces (i.e. general structural similarity), and *PrISE_C _*combines local structural similarity and general structural similarity to predict interface residues. The performances of these three predictors were compared using the DS188 dataset. For this experiment, samples were extracted from the *ProtInDB *repository. In addition, samples extracted from proteins sharing more than 95% of sequence identity with the query protein and belonging to the same species were excluded from the prediction process to avoid overestimation on the predictions. To simulate a random prediction, the interface/non-interface labels associated with the central residue in each sample in the repository were randomly shuffled. The results of this experiment are presented in Figure 2 as precision-recall curves. These results indicate that *PrISE_L_, PrISE_G_*, and *PrISE_C _*outperform the random predictor. Furthermore, *PrISE_C _*achieves similar or better performance than *PrISE_G _*whereas *PrISE_G _*predictions are superior to those of *PrISE_L_*. Similar conclusions are supported by experiments using the DS24Carl, DS56bound and DS56unbound datasets ^*d*^. As a consequence, *PrISE_C _*was selected to perform the experiments presented in the next subsections.

### Impact of homologs of the query protein on the quality of predictions

We assess the extent to which the predictions are impacted by the presence of structural elements derived from sequence homologs of the query protein. The first experiment excludes samples derived from proteins belonging to the same species that share ≥ 95% of sequence identity with the query protein (called *homologs from the same species*). The second experiment excludes samples from all the proteins that share ≥ 95% of sequence identity with the query protein (referred to as *homologs*).

**Figure 3:**
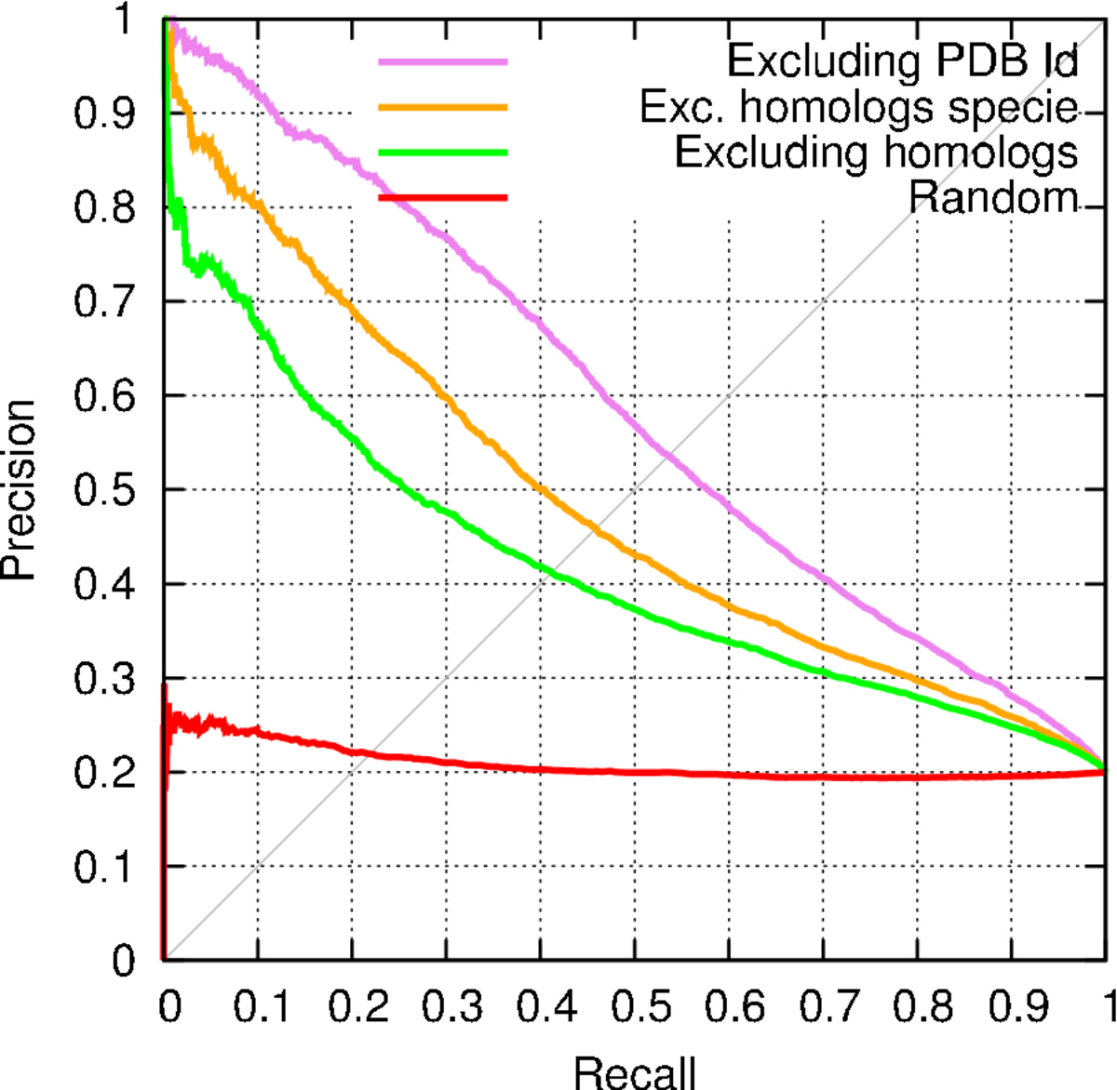
**Comparison of schemes for filtering out similar proteins from the prediction process**. This experiment was performed using PrISE_C _with the DS188 dataset.

Figure 3 compares the two methods for excluding homologs with a setup in which only the samples derived from proteins with the same PDB ID as the query proteins are excluded ^*e*^. As seen from Figure 3, the prediction performance is better when sequence homologs of the query protein are not excluded from the set of proteins used to generate the repository used for making the predictions. The best performance is achieved by excluding the proteins with the same PDB ID as those of the query proteins.

### Comparison with two prediction methods based on geometric-conserved local surfaces

We compared the three predictors from the *PrISE *family with the predictors proposed by Carl et al. in [37,41]. These methods rely on conservation of the geometry and the physico-chemical properties of surface patches to predict interfaces. In [37], the conserved regions were extracted from proteins with similar structures. In [41], similar performance was achieved using conserved regions extracted using local structural alignments. This comparison was performed using the DS24Carl dataset composed of 24 proteins and generated in [41]. In the case of the *PrISE *family of methods, samples were retrieved from the *ProtInDB *repository. Samples extracted from proteins sharing more than 95% of sequence identity with the query protein and belonging to the same species were not used in the prediction process. The results of the experiment, presented in Table 1, indicate that each of the three predictors from the *PrISE *family outperforms the predictors described in [37,41]. The differences in performances may be explained by the differences in the prediction techniques. In particular, *PrISE *family of predictors, unlike those of Carl et al., exploit the interface/non-interface labels associated with surface patches that share structural similarity with the surface neighborhood of each surface residue of the query protein.

**Table 1:** Performance of different methods on the DS24Carl dataset

Predictor	Precision%	Recall%	F1%	Accuracy%	CC%	AUC%
Carl08	31.5	35.3	33.3	-	-	-
Carl10	32.0	34.0	33.0	-	-	-
*PrISE_L_*	45.1	56.2	50.0	69.1	27.1	70.5
*PrISE_G_*	53.9	58.7	56.2	75.1	36.8	75.6
*PrISE_C_*	58.3	58.3	58.3	77.5	40.6	77.1

Performance measures are computed as the average on the set of 24 proteins. Precision and recall values for Carl08 and Carl10 were taken from [37] and [41] respectively.

Results of a similar experiment excluding samples extracted from homologs of the query proteins, as well as results of experiments using the *protInDb *⋂ *PQS *repository, are presented in section six of the Additional File 1.

### Comparison with a prediction method based on protein structural similarity

We compared *PrISE_C _*with *PredUs *[38,39], a method that relies on protein structural similarity, using the DS188, DS56bound and DS56unbound datasets. *PredUs *is based on the idea that interaction sites are conserved among proteins that are structurally similar to each other. *PredUs *computes a structural alignment of the query protein with every protein in a set of proteins with known interface residues. The alignments are used to extract a *contact frequency map *which indicates for each residue in the query protein, the number of interface residues that are structurally aligned with it. The contact frequency map is then used to predict whether each residue on the query protein is an interface residue. In [38], the prediction was performed using a logistic regression function that receives as inputs the counts contained in the contact frequency maps. In [39], the logistic regression function was replaced by a support vector machine (SVM) classifier that uses accessible surface areas and the counts contained in the contact frequency maps to perform prediction.

In order to perform a fair comparison between *PrISE *and *PredUs*, the structural elements used by *PrISE *and the structural neighbors used by *PredUs *were extracted from the same dataset of proteins. This dataset corresponds to the subset of proteins that are common to both *ProtInDB *and PQS which ensures the largest overlap between the proteins used by *PredUs *(which relies on the structural neighbors extracted from the PDB and PQS) and *PrISE *(which relies on the proteins extracted from biological assemblies in the PDB and deposited in *ProtInDB*). This resulting dataset, used to create the *protInDB*∩ *PQS *repository, includes 55,974 protein chains derived from 21,786 protein complexes. *PredUs *predictions were obtained from the available web server [39]. This server allows us to choose the set of structural neighbors to be considered in the prediction process. Using this feature, we were able to exclude from the sets of structural neighbors those proteins that were not in the intersection of *ProtInDB *and PQS as well as homologs or homologs from the same species.

**Figure 4:**
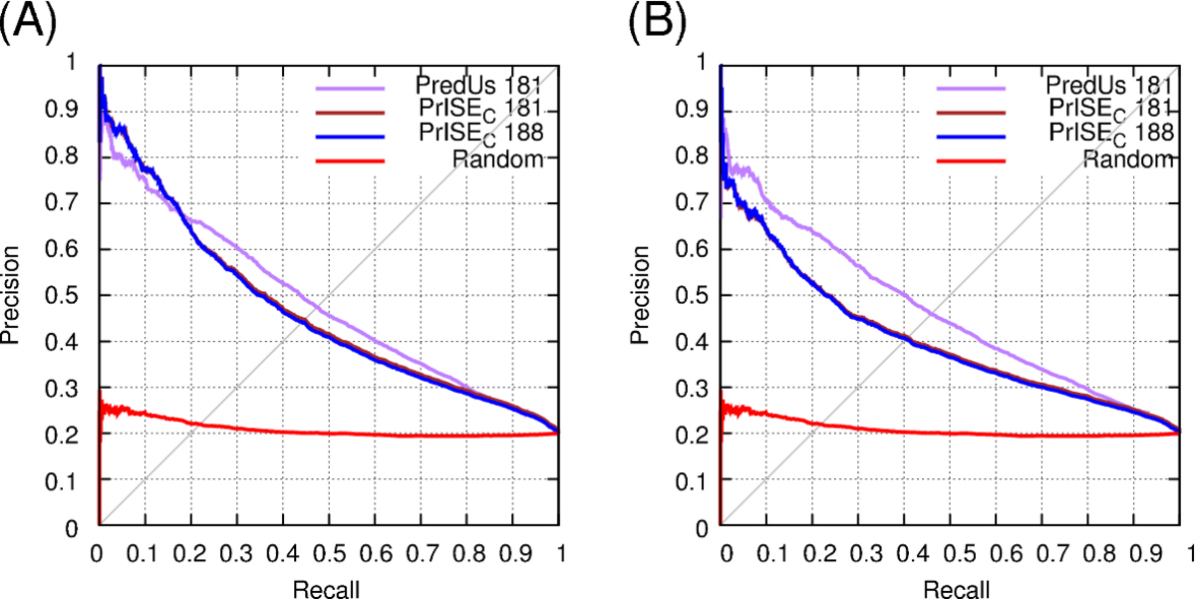
**Comparison of PredUs and PrISE_C _using the dataset DS188, derived from the docking benchmark 3.0**. (A) performance of predictions from which homologs from the same species were not used to compute the structural neighbors and the samples used in *PredUs *and *PrISE *respectively. (B) performance of predictions that did not consider homologs. Both images show results for the 181 proteins that were predicted by *PredUs *and *PrISE_C _*and for the 188 proteins predicted by *PrISE_C_*.

A first comparison of the *PrISE *family of predictors and *PredUs *was carried out using the DS188 dataset. However, since the SVM used by *PredUs *was trained using this dataset [39], it is likely that the estimated performance of *PredUs *in this case is overly optimistic, resulting in an unfair comparison with *PrISE*. We found that in 7 of 188 cases (corresponding to the PDB Ids and chains 1ghq-A, 1gp2-G, 1t6b-X, 1wq1-G, 1xd3-B, 1z0k-B, and 2ajf-A) *PredUs *failed to find structural neighbors, and hence failed to predict interfaces. In contrast, the *PrISE *predictors found the structural elements needed to produce predictions for the 188 cases. Predictions including these seven cases are labeled as *PrISE_C _*188 in Figure 4, whereas predictions of *PrISE_C _*and *PredUs *considering the set of 181 proteins are labeled with the suffix 181. The performances of *PrISE_C _*in the two cases are similar. *PredUs *generally outperforms *PrISE_C_*, the best performing predictor from the *PrISE *family. This result is not surprising given that the SVM used by *PredUs *was trained on this dataset whereas *PrISE *did not have this advantage.

**Figure 5:**
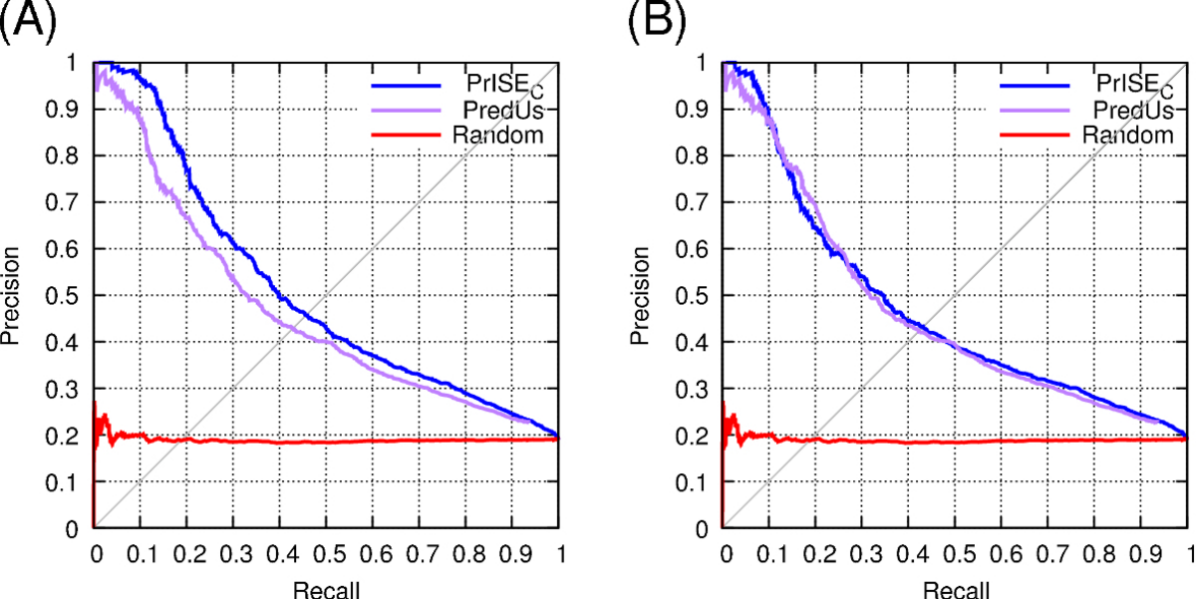
**Comparison of PrISE_C _and PredUs using the dataset DS56bound, derived from CAPRI**. The results in (A) correspond to predictions in which homologs from the same species were excluded from the collection of samples and the set of structural neighbors. The results in (B) were obtained excluding homologs from the sets of similar structures.

A second comparison of *PrISE_C _*and *PredUs *was performed using the DS56bound dataset. *PrISE_C _*and *PredUs *generated predictions for all the proteins in this dataset. The precision-recall curves presented in Figure 5 show that when homologs from the same species are excluded from the collection of similar structures, *PrISE_C _*outperforms *PredUs*, but when homologs are excluded regardless of the species, the performances of *PrISE_C _*and *PredUs *are comparable. These results indicate that the use of local surface structural similarity is a competitive alternative to the use of protein structural similarity for the problem of predicting protein-protein interface residues.

An evaluation considering additional performance measures is presented in Table 2. The data in this table indicates that *PrISE_C _*outperforms *PredUs *in terms of F1, correlation coefficient, or area under the ROC. The values for precision, recall, F1, Accuracy and CC were computed using the default cutoff values for *PrISE_C _*and *PredUs*.

**Table 2:** Evaluation of PrISE_C _and PredUs on DS56bound using different performance measures

Filter out	Predictor	Precision%	Recall%	F1%	Accuracy%	CC%	AUC%
**Homologs from the same species**	*PredUs*	44.3	39.8	41.9	80.4	30.2	75.1
	*PrISE_C_*	46.1	45.4	45.7	80.9	34.1	77.6

**Homologs**	*PredUs*	44.5	38.5	41.3	80.6	29.8	74.9
	*PrISE_C_*	43.6	42.4	43.0	80.0	30.9	76.3

The table is divided into two sections depending on which proteins are excluded from the set of similar structures (First column)

**Figure 6:**
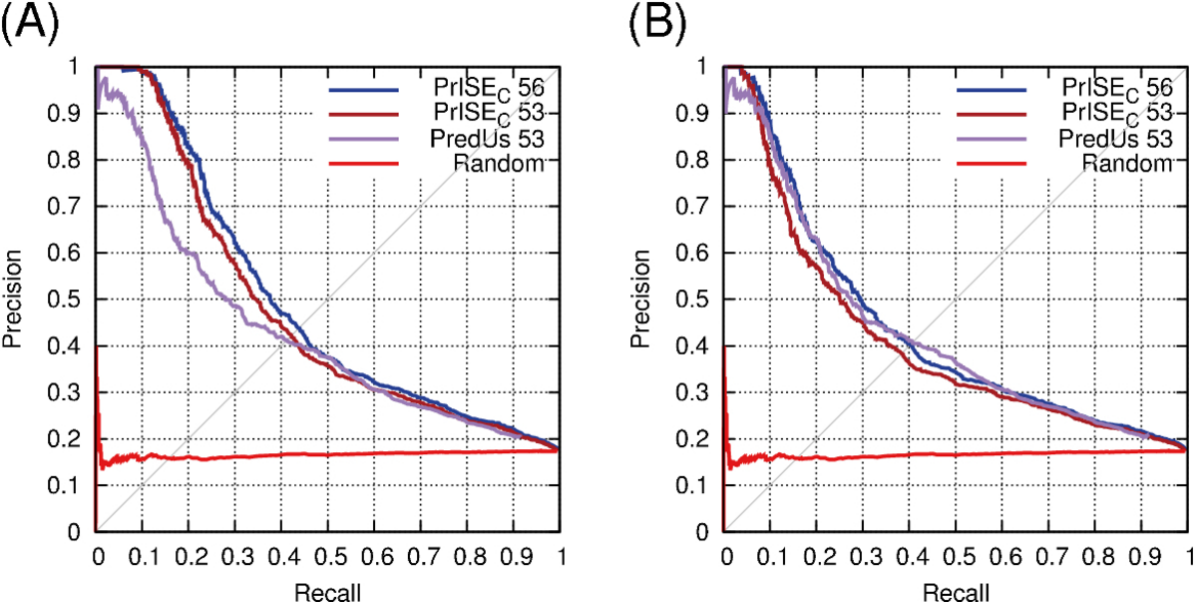
**Comparison of PrISE_C _and PredUs using the DS56unbound dataset, derived from CAPRI**. (A) shows the performance achieved after removing homologs from the same species from the set of similar structures in *PredUs *and *PrISE_C_*. (B) shows the performances when homologs are excluded. The suffixes 53 and 56 indicate the number of proteins that were used in the experiment.

A final comparison between *PrISE_C _*and *PredUs *was performed using the DS56unbound dataset. Three out of the 56 proteins (corresponding to the PDB IDs-chains 1ken-H, 1ken-L, and 1ohz-B) were not processed by *PredUs *because no structural neighbors were found. Figure 6 shows the precision-recall curves of *PrISE_C _*and *PredUs *on the 53 cases covered by *PredUs*, as well as the performance of *PrISE_C _*when all the 56 proteins are considered. A comparison of both predictors using the set of 53 proteins and excluding homologs from the same species indicates that *PrISE_C _*outperforms *PredUs *for precision values > 0.4. On the contrary, when homologs are excluded, the performance of *PredUs *is better than the performance of *PrISE_C _*for precision values ≥ 0.3. Finally, the performance of *PrISE_C _*computed on 56 proteins is, surprisingly, slightly better than the performance computed on 53 proteins. This suggests that local structural similarity based interface prediction methods can be effective even in the absence of globally similar structures.

An evaluation of *PrISE_C _*and *PredUs *using additional performance measures is presented in Table 3. *PrISE_C _*outperforms *PredUs *in terms of F1, CC and AUC when homologs from the same species are excluded from the set of similar structures. When homologs are excluded, *PredUs *outperforms *PrISE_C _*on the set of 53 proteins predicted by *PredUs*.

**Table 3:** Evaluation of PrISE_C _and PredUs on DS56unbound using different performance measures

Filter out	Predictor	Precision%	Recall%	F1%	Accuracy%	CC%	AUC%
**Homologs from the same species**	*PredUs *53	43.2	37.2	39.9	81.8	29.4	73.6
	*PrISE_C _*53	42.3	42.1	42.2	81.2	31.0	74.8
	*PrISE_C _*56	43.7	44.0	43.8	81.2	32.6	75.5

**Homologs**	*PredUs *53	42.6	36.8	39.5	81.6	28.8	73.5
	*PrISE_C _*53	38.8	37.9	38.4	80.1	26.5	72.9
	*PrISE_C _*56	40.5	40.0	40.2	80.2	28.4	73.7

### Comparison with other prediction methods

We compared the performances of *PrISE_C_*, Promate [25], PINUP [48], Cons-PPISP [49], and Meta-PPISP [50] using all the proteins in the DS56bound and DS56unbound datasets. The choice of the predictors used in this comparison was based on the results of a comparative study in which they were reported to achieve the best performance among the six different classifiers on two different datasets [8]. Promate uses a scoring function based on features describing evolutionary conservation, chemical character of the atoms, secondary structures, distributions of atoms and amino acids, and distribution of b-factors. Cons-PPISP's predictions are based on a consensus between different artificial neural networks trained on conservation sequence profiles and solvent accessibilities. PINUP uses an empirical scoring function based on side chain energy scores, interface propensity and residue conservation. Meta-PPISP uses linear regression on the scores produced by Cons-PPISP, Promate and PINUP.

In the experiments presented in this subsection, we considered the performance of two *PrISE_C _*classifiers according to which proteins were filtered out from the process of extraction of samples: homologs from the same species as the query protein and homologs regardless of the species. The scores used to generate the precision-recall curves of Promate, PINUP, Cons-PPISP and Meta-PPISP were computed using Meta-PPISP's web server.

**Figure 7:**
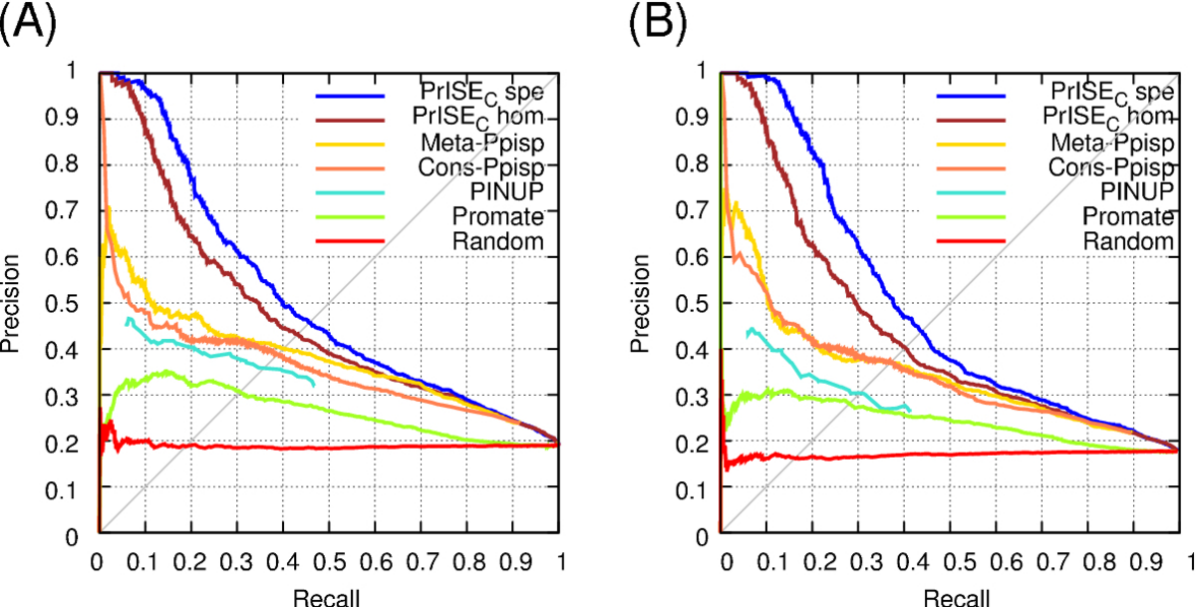
**Performance of different classifiers evaluated on the DS56bound (A) and the DS56unbound (B) datasets**. For the *PrISE *classifiers, "spe." and "hom." show predictions in which samples extracted from homologs from the same specie and homologs, respectively, has been excluded from the prediction process.

The precision-recall curves corresponding to the evaluation of the classifiers on the DS56bound and DS56Unbound datasets are shown in Figure 7. On both datasets, *PrISE_C _*predictors outperform Meta-PPISP for precision values > 0.35 and achieve performance comparable to that of Meta-PPISP for precision values ≤ 0.35. Furthermore, *PrISE_C _*outperform Promate, PINUP, and Cons-PPISP over the entire range of precision and recall values.

An evaluation considering additional performance measures is presented in Table 4. All the performance measures, with exception of AUC ROC, were computed using threshold values of 0.56, 0.28, 0.41, 0.34, and 0.34 on the scores generated by Promate, PINUP, Cons-PPISP, Meta-PPISP, and *PrISE_C _*respectively. These threshold values correspond to the default values defined in the Meta-PPISP and *PrISE_C _*web servers. The results show that the *PrISE_C _*predictors outperform the other predictors on both datasets in terms of F1, correlation coefficient and area under the ROC.

**Table 4:** Evaluation on the datasets DS56bound and DS56unbound

Dataset	Predictor	Precision%	Recall%	F1%	Accuracy%	CC%	AUC%
	Promate	31.9	27.3	29.4	76.7	15.6	63.3
	PINUP	37.3	31.9	34.4	78.4	21.7	63.7
**DS56bound**	Cons-PPISP	39.8	36.1	37.9	78.9	25.2	72.6
	Meta-PPISP	43.3	25.8	32.3	80.8	22.9	74.4
	*PrISE_C _*spe.	46.1	45.4	45.7	80.9	34.1	77.6
	*PrISE_C _*hom.	43.6	42.4	43.0	80.0	30.9	76.3

	Promate	28.7	27.3	28.0	76.6	14.0	62.7
	PINUP	30.4	30.1	30.2	76.9	16.4	60.0
**Ds56unbound**	Cons-PPISP	37.4	34.5	35.9	79.5	23.8	71.2
	Meta-PPISP	38.9	24.0	29.7	81.1	20.2	71.5
	*PrISE_C _*spe.	43.7	44.0	43.8	81.2	32.6	75.5
	*PrISE_C _*hom.	40.5	40.0	40.2	80.2	28.4	73.7

"*PrISE_C _*spe." refers to the performance computed after filtering out from the repository samples extracted from homologs from the same species. "*PrISE_C _*hom." indicates that samples extracted from homologs were not considered in the prediction process.

**Figure 8:**
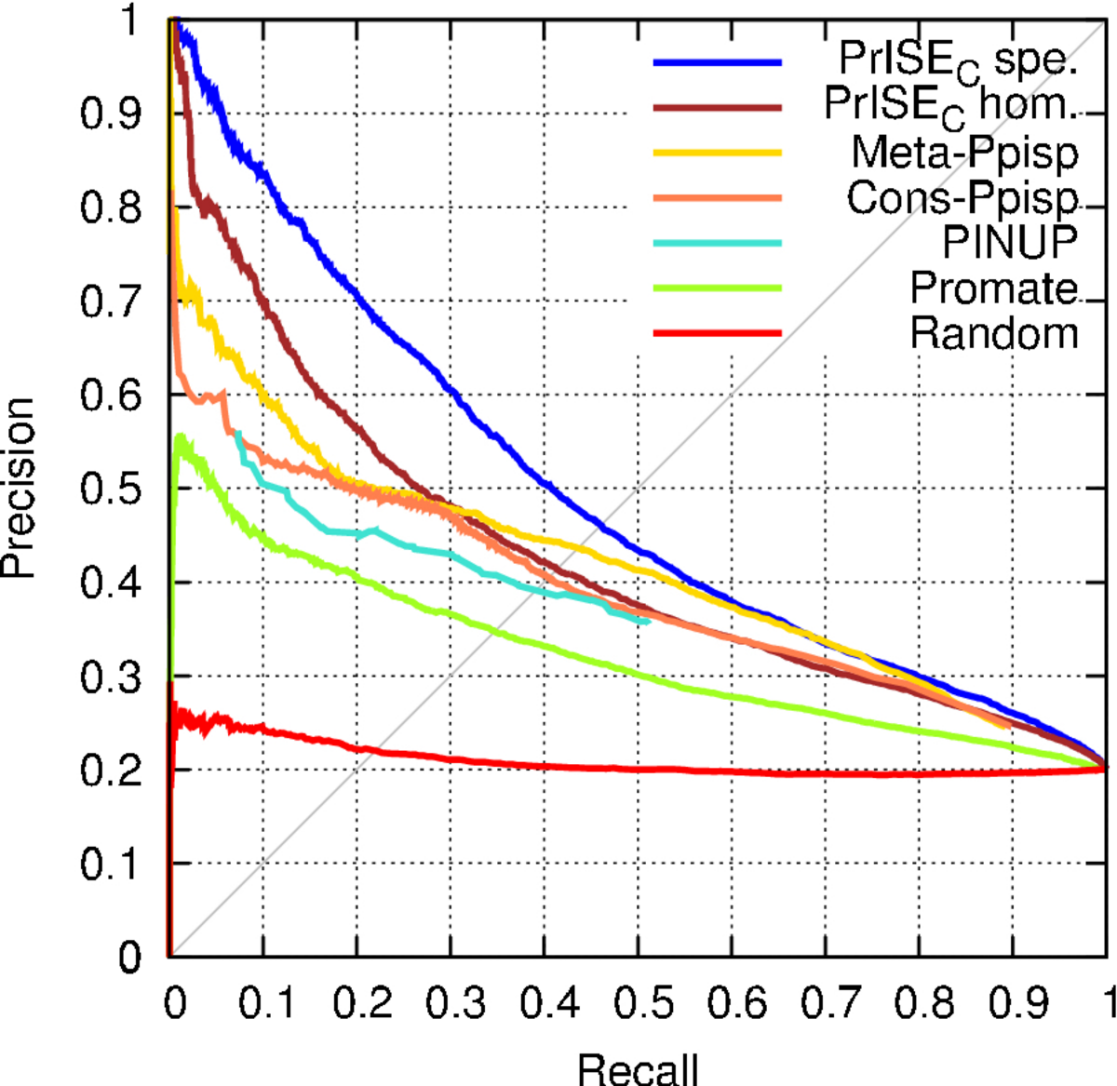
**Precision-recall curves of different classifiers evaluated on 187 proteins from the DS188 dataset**. For the PrISE classifiers, "spe." and "hom." show predictions in which homologs from the same species and homologs, respectively, has been excluded from the repository of structural elements.

The results of an experiment using 187 proteins from the DS188 dataset are presented in Figure 8. Protein chain 2vis-C was excluded from the experiment given that Promate could not generate a prediction. When homologs from the same species are excluded, *PrISE_C _*outperforms the other predictors except Meta-PPISP. *PrISE_C _*outperforms Meta-PPISP for precision values > 0.4 and achieves comparable performance to that of Meta-PPISP for precision values ≤ 0.4. When homologs are excluded, the performance of *PrISE_C _*is superior that the performance of PINUP and Promate. *PrISE_C _*outperforms Meta-PPISP and Cons-PPISP for precision values > 0.5, and is outperformed by Meta-PPISP for precision values ≤ 0.45.

An evaluation using different performance measures is presented in Table 5. According to this table, the performance of both *PrISE *predictors is superior that the performance of the other classifiers in terms of F1 and CC. Furthermore, when homologs from the same species are excluded, *PrISE_C _*outperforms the other classifiers in terms of AUC.

**Table 5:** Evaluation on 187 proteins from DS188

Predictor	Precision%	Recall%	F1%	Accuracy%	CC%	AUC%
Promate	36.5	30.3	33.1	77.1	19.5	67.7
PINUP	40.7	34.7	37.5	78.3	24.6	66.0
Cons-PPISP	46.5	30.6	36.9	80.4	26.7	73.2
Meta-PPISP	49.0	26.7	34.6	81.1	26.2	74.6
*PrISE_C _*spe.	48.0	43.2	45.5	80.6	33.8	77.2
*PrISE_C _*hom.	43.2	38.1	40.5	79.0	27.9	74.2

"PrISE_C _spe." refers to the performance computed after excluding from the prediction process samples extracted from homologs of the same species that the query proteins. "PrISE_C _hom." indicates that samples extracted from homologs were filtered out from the repository

### Prediction performances in the absence of similar proteins

**Figure 9:**
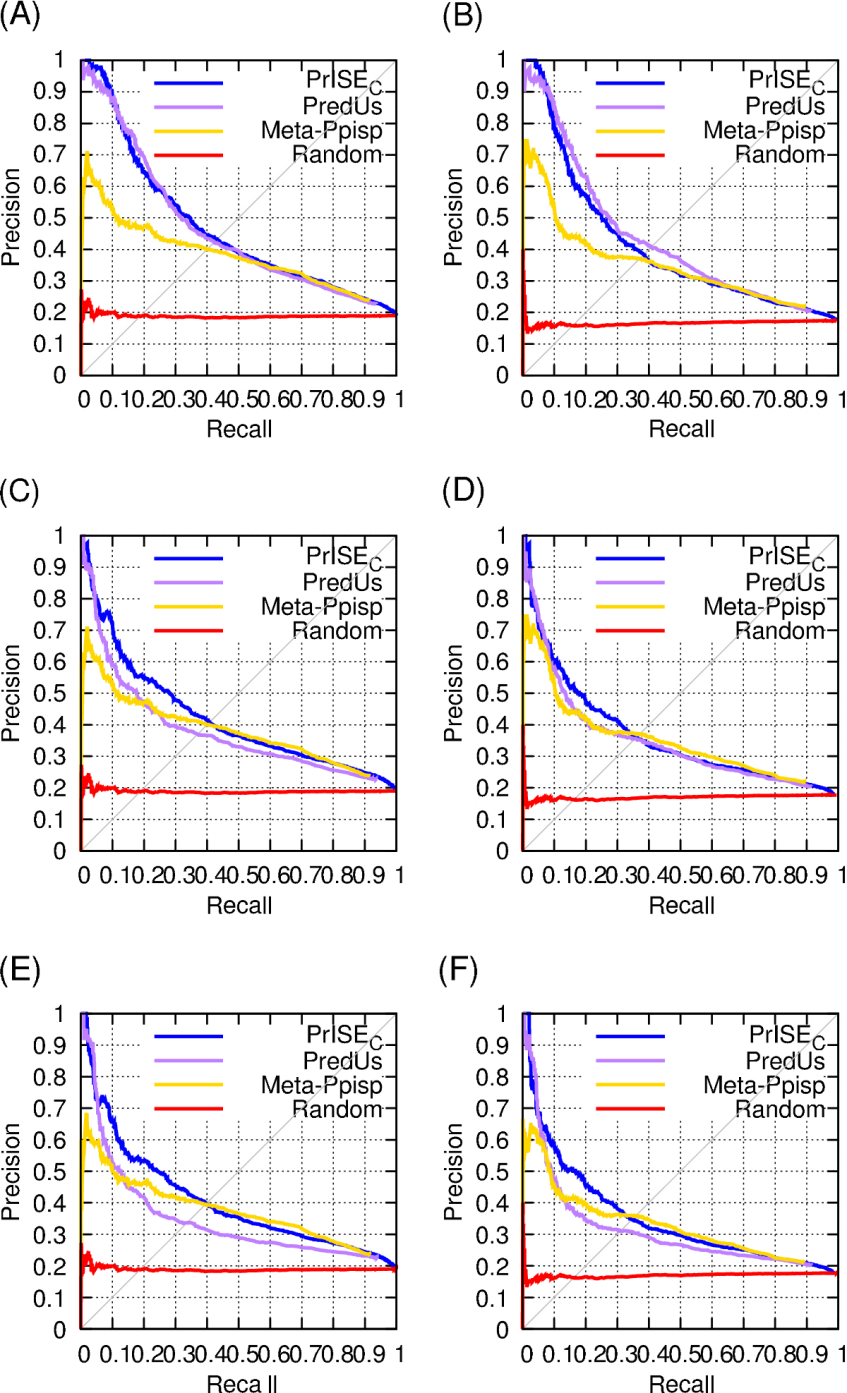
**Performance computed in absence of similar proteins at different similarity levels**. Figures (A) and (B) show the precision recall curves computed after excluding from the sets of similar structures homologs (without regarding the species) sharing ≥ 95% of sequence identity with the query proteins. Similarly, figures (C) and (D) show the performances after excluding proteins sharing ≥ 50% sequence identity, and (E) and (F) display the results after filtering out proteins with sequence identity ≥ 30%. The precision-recall curves corresponding to the DS56bound dataset are shown at (A), (C), and (E), and the results computed using the DS56Unbound dataset are labeled as (B), (D), and (F). Figures (E) and (F) were computed using 55 and 52 proteins respectively given that PredUs could not find structural elements for the protein chain 1ynt-L.

To evaluate the extent to which the performances of *PrISE_C _*and *PredUs *depend on the degree of homology between the query proteins and the proteins used to extract samples or structural neighbors, we compare the results obtained using three different sequence homology cutoffs: 95%, 50% and 30%. The results, shown in Figure 9, indicate that *PredUs *is more sensitive than *PrISE_C _*to the lack of similar proteins in the sets used to extract similar structures. The figure also shows that the performance of *PrISE_C _*is competitive with that of Meta-PPISP even when the repository used by *PrISE_C _*is composed by proteins sharing < 30% of sequence identity with the query proteins.

## Conclusions

We have shown that it is possible to reliably predict protein-protein interface residues using only local surface structural similarity with proteins with known interfaces.

The experiments comparing the performance of the *PrISE *family of predictors with the structural similarity based interface predictors of Carl et al. [37,41] show that the use of interface/non interface labels of residues in structurally similar surface patches leads to improved predictions by *PrISE*. This observation is also supported by the results obtained using *PredUs*, that implicitly exploits information about non-interface residues reflected in the contacting frequencies of interface residues.

Surface structural similarity based methods for interface residue prediction may use local similarity, overall similarity, or a combination of both. *PrISE_L_*, which relies on the similarity between structural elements (i.e. local structural similarity), outperforms random prediction; *PrISE_G _*which relies on the similarity between protein surfaces (i.e. general structural similarity) outperforms *PrISE_L_*. This result may not be surprising in light of the influence that regions outside the immediate local environment have on the conformation of protein complexes. However, our results show that the best predictions are achieved by *PrISE_C_*, using a combination of local and overall surface similarity.

Our results indicate that, in general, *PrISE_C _*outperforms several state of the art predictors such as Promate, PINUP, Cons-PPISP, and Meta-PPISP. Blind comparisons of *PrISE_C _*and *PredUs *using the same proteins to extract samples and structural neighbors respectively, indicate that *PrISE_C _*achieves performance that is superior to or comparable with that of *PredUs*. Furthermore, *PrISE_C _*is more robust that *PredUs *at low levels of homology between the query proteins and proteins in the sets used to extract similar structures, while remains competitive with Meta-PPISP.

The interface residue prediction methods such as *PrISE *that use only local surface structural similarity have an advantage relative to methods that rely on global structural similarity: The former can produce predictions whereas the latter cannot in the absence of protein with structures that are sufficiently similar to the structure of the query protein.

Another advantage of the *PrISE *family of predictors is that the information needed to compute similar structural elements (i.e. residues in the structural elements, accessible surface area of these residues and their histogram of atom nomenclatures) can be obtained in a reasonable amount of time. The time required for retrieving the samples associated with a query protein from a repository of 21,289,060 structural elements extracted from 88,593 protein chains is in average 90 seconds using a personal computer (Intel Core2 Duo CPU at 2.40 GHz, 4 MB of RAM and a hard disk of 232 GB).

We conclude that methods based on local surface structural similarity are a simple yet effective approach to the problem of prediction of protein-protein interface residues.

## Endnotes

a. An explanation of the process used to select the city block metric from a set of different metrics is presented in the Additional File 1.

b. Based on results of exploratory experiments, we found that 50, 200, and 500 similar structural elements are adequate (respectively) for performing prediction using *PrISE_L_, PrISE_G_*, and *PrISE_C_*. See Figures 4 to 6 and the corresponding discussion in the Additional File 1 for details.

c. See the Additional File 1 for a discussion on the choice of the threshold.

d. See section four of the Additional File 1, that also includes an example of the relationship between the scores of the predictors in the *PrISE *family.

e. Additional results using DS24Carl, DS56bound and DS56unbound are presented in section five of the Additional File 1.

## Competing interests

The authors declare that they have no competing interests.

## Authors' contributions

The study was originally conceived by VH and RAJ. RAJ carried out the experiments. All the authors discussed the experimental design, and participated in the analysis and interpretation of the data. RAJ wrote the initial draft of the manuscript. All authors revised and approved the final manuscript.

## Supplementary Material

Additional file 1 **Supplementary information**. Click here for file
