# Supplementary material for: Predicting protein-protein interface residues using local surface structural similarity
Source: BMC Bioinformatics. 2012 Mar 18;13:41. doi: 10.1186/1471-2105-13-41 (PMC3386866; doi:10.1186/1471-2105-13-41)
Supplement: Additional file 1 — Supplementary information. [file 1471-2105-13-41-S1.PDF]

Supplementary information
Predicting protein-protein interface residues using local surface structural similarity

This document provides additional information about the process used for building the $PrISE$ family of predictors of protein-protein interface residues as well as supplementary results of some of the experiments included in the companion paper. The first two sections describe details about the histograms of atom nomenclatures and the constraints used to retrieve similar structural elements from a repository of structural elements. The next section describe the dataset and the experiments used for tuning the parameters of $PrISE_G$, $PrISE_L$, and $PrISE_C$. The remaining sections show the results of complementary experiments to the reported in the companion paper performed on different datasets.

1 Atom nomenclatures.

A list of the 36 atom nomenclatures used to build the histograms of atom nomenclatures (HAN) is presented in Table 1. These nomenclatures were extracted from PDB.

C	CA	CB	CD	CD1	CD2
CE	CE1	CE2	CE3	CG	CG1
CG2	CH2	CZ	CZ2	CZ3	N
ND1	ND2	NE	NE1	NE2	NH1
NH2	NZ	O	OD1	OD2	OE1
OE2	OG	OG1	OH	SD	SG

Table 1: Atom nomenclatures used to build the histograms of atom nomenclatures.

2 Retrieving similar structural elements - additional details

As explained in the methods section of the companion paper, we defined four constraints that every structural element retrieved from a repository should comply to be considered similar to a query structural element:

> "(i) q_r and q_s must not be from the same protein complex; (ii) the central residues r and s of the structural elements q_r and q_s respectively, must be identical; (iii) the difference between the accessible surface areas of r and s should be $\leq 5\%$ of the maximum accessible surface area of residues identical to r; and (iv) the differences between the accessible surface areas of q_r and q_s must be $\leq 15\%$ of the maximum estimated accessible surface area of any structural element centered on a residue identical to r".

Constraint (iii) requires the computation of the difference between the accessible surface area of the central residues r and s of two structural elements q_r and q_s respectively. This difference, denoted by $dASAres$, is computed as:

$$dASAres(r, s) = \frac{|asaRes(r) - asaRes(s)|}{maxAsaRes(r) - minAsaRes(r)} \times 100\%$$

where $asares(r_1)$ denotes the accessible surface area of the residue r_1, and $minAsaRes(r_1)$ and $maxAsaRes(r_1)$ denotes the experimental minimum and maximum accessible surface area of the residue r_1 respectively[1]. The values of $maxAsaRes$ and $minAsaRes$ were estimated from a dataset of 400 proteins randomly selected from ProtInDb [2], a database of protein-protein interface residues. the lower the values of $dASAres$, the highest the similarity between the accessible surface areas of the residues r and s.

Constraint (iv) requires the computation of the difference between the accessible surface areas of two structural elements q_1 and q_2. This difference, denoted by $dASAse$, is computed as:

$$dASAse(q_1, q_2) = \frac{|asaSe(q_1) - asaSe(q_2)|}{maxAsaSe(q_1) - minAsaSe(q_1)} \times 100\%$$

where $asaSe(q)$ denotes the summation of the accessible surface area of the surface atoms in the structural element q. An atom is considered to be a surface atom if its accessible surface area is > 0 Å^2. $MinAsaSe(q)$ and $maxAsaSe(q)$ represent the estimated minimum and

[1] Note that according to constraint (ii) residue r is identical to residue s.

[2] http://protindb.cs.iastate.edu

maximum accessible surface areas of structural elements centered on a residue identical to the central residue of q. These two values were estimated from the dataset of 400 proteins extracted from ProtInDb. The interpretation of $dASAse$ is similar to the interpretation of $dASAres$ (i.e. the lowest the value of $dASAse(q_1, q_2)$, the highest the similarity between the accessible surface areas of the structural elements q_1 and q_2).

3 Tuning method

We tuned the parameters of the $PrISE$ family of predictors in two steps. The goal of the first step was to efficiently retrieve structural elements from the repository of structural elements for all the structural elements in a query protein. The goal of the second step was to maximize the prediction performance. We use the ProtInDb repository of structural elements to perform these experiments.

3.1 Tuning dataset

The tuning data set is composed of 50 chains (see Table 2) with more than 40 residues, resolution $\leq$ 2.5 Å, and sequence identity $\leq$ 15%. This dataset has 10,379 residues from which 1,946 are interface residues.

1df4A	1risA	2dkoB	2qeeA	3fedA
1dqzA	1s72H	2dw5A	2vn6A	3h7hB
1dysA	1smxA	2hdiB	2vtbA	3hf5A
1euvA	1t0bA	2iihA	2ww2A	3hm4A
1i2cA	1u5kA	2izzA	2xdpA	3k94A
1j34C	1u9dA	2jkhL	2zewA	3kb4A
1kqfC	1uuyA	2o2vA	3ag3I	3kz5B
1kyfA	1v05A	2o70A	3bm3A	3m9lA
1pytA	1yrkA	2odeA	3ct6A	3mcwA
1q7lB	2cchB	2pmuA	3d32B	3pg6A

Table 2: List of the 50 protein chains included in the tuning dataset.

3.2 Representative set of similar structural elements

We wanted to efficiently obtain a set of similar structural elements (samples) from the repository that allows us to perform predictions for all the structural elements in a query protein. To achieve this goal, we performed a grid search using values equivalent to 5%, 10% and 15% on the parameters $dASAres$ and $dASAse$. We found that using $dASAres \leq 5\%$ and $dASAse \leq 15\%$ we can retrieve samples for all the structural elements in the dataset. The threshold of 5% on $dASAres$ allows us to obtain samples whose central residues are as similar as possible to the central residue of a query structural element. The threshold of 15% on $dASAse$ allows us to include some flexibility to account for conformational changes in residues in the fringe of the structural elements whereas minimizes the potential problem of lack of samples for query proteins not included in the tuning dataset.

3.3 Performance tuning

We analyzed the impact of different factors in the performance of the $PrISE$ family of predictors that extracted samples with dASAres $\leq$ 5%, and dASAse $\leq$ 15%. We evaluated several metrics of distance between histogram of atom nomenclatures as well as several schemes used to assign weights to the samples and to find the number of samples that maximized the performance of the predictions.

Evaluation of distance metrics for histogram of atom nomenclatures

We evaluated six different metrics of distance between histograms selected from a survey presented in [3]: Inner product, fidelity, Euclidean distance, city block distance, symmetric Kullback–Leibler divergence, and symmetric Kullback–Leibler divergence with Laplace estimates[3]. We predicted a residue as an interface if the majority of the central residues of the top 50 samples (according to each metric) are interface residues. The results of these experiments, presented as precision-recall curves in Figure 1, indicate that predictions using the city block and the Euclidean metrics outperform predictions using the other metrics. However the performance achieved using city block distance is slightly better that the same using Euclidean distance in the central part of the curves. Hence, we selected the city block metric to compute the distances between histogram of atom nomenclatures (DHAN).

Evaluation of different schemes to assign weights to the samples

We performed several experiments to evaluate different alternatives to assign weights to the samples and to find an adequate number of samples that maximized the performance of the prediction in the tuning dataset. These experiments were performed with dASAres $\leq$ 5%, dASAse $\leq$ 15%, and using the city block metric for comparing distances between histograms of atom nomenclatures.

To set a base case for the comparisons presented in this subsection, we performed predictions using majority vote

[3] The Laplace estimates add 0.0001 to each bin of the HAN. This allows to perform comparisons between empty and non-empty bins in the histograms of the query structural element and a sample using Kullback–Leibler divergence.

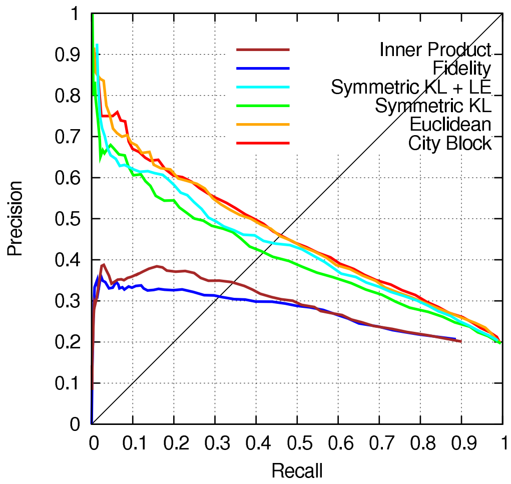

Figure 1: Prediction results using majority vote on the top 50 samples according to different definitions of distance between histogram of atom nomenclatures.

on the top n unweighted samples according to the ordering determined by the values of DHAN. The results of these experiments, shown in Figure 2, indicate that the prediction performance is not significantly affected by the number of unweighted samples.

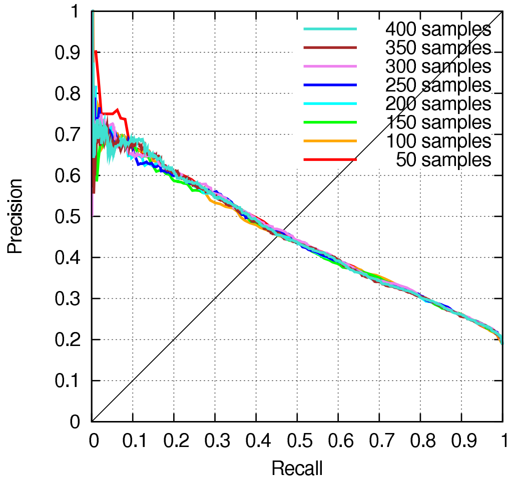

Figure 2: Prediction results using majority vote with different number of unweighted samples.

A second experiment was performed using majority vote on samples weighted using the normalized DHAN as:

$$w(s,q) = 1 - \frac{DHAN(s,q)}{\max_{r \in S(Q), q_1 \in S_r,} \{DHAN(q_1, r)\}}$$

where s is a sample associated with the query structural element q, $S(Q)$ represents the set of all the structural elements of a query protein Q, and S_r represents the set of all the samples associated with a query structural element r. The normalization term corresponds to the largest $DHAN$ between any structural element in a query protein and its associated samples. Hence, samples with lower DHAN values are assigned larger weights. The results of this experiment, presented in Figure 3, indicate that the best performance was achieved using the top 20 to 30 samples. A comparison between these results and the results presented in Figure 2 shows that the best performance was achieved when the samples were weighted using DHAN.

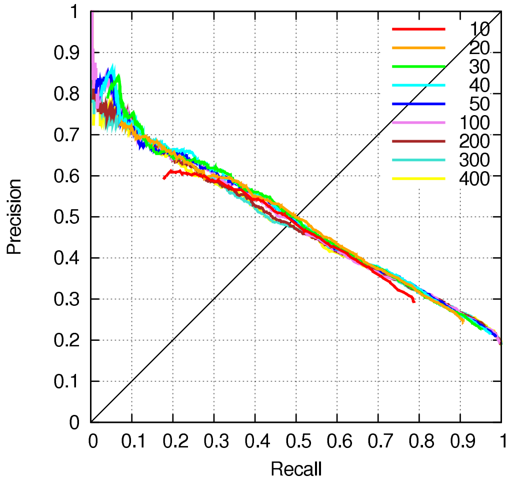

Figure 3: Prediction results with different number of samples and using majority vote on samples weighted using the city block distance between histogram of atom nomenclatures.

The following experiments evaluated the weighting schemes proposed for each member of the $PrISE$ family of predictors. For $PrISE_G$, the weight of each sample extracted from protein p (described by equation (1) in the methods section in the paper) is computed as the total number of samples extracted from p. Hence, samples extracted from proteins with higher general structural similarity to the query protein (according to the number of samples) are assigned larger weights. For $PriSE_L$ (see equation (2) in the paper), the weight of a sample extracted from protein p is computed as the number of samples extracted from p that are associated with the structural elements in a region surrounding the query structural element (i.e. local similarity). The $PrISE_C$ predictor (equation (3) in the paper), weights each sample using information derived from the combination of local and general similarity.

The results of an evaluation of $PrISE_G$ using different number of samples are presented in Figure 4. These results indicate that the best prediction was achieved using 100 to 200 samples.

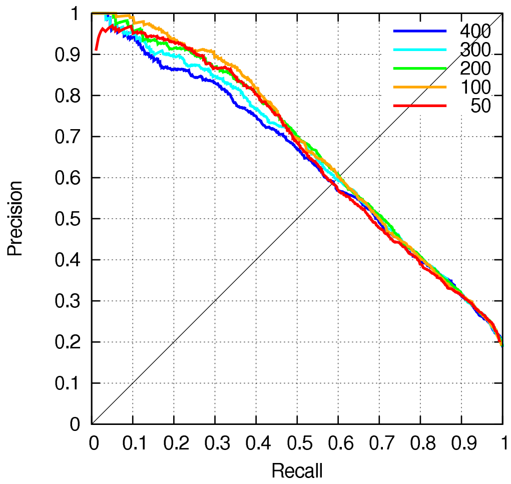

Figure 4: Prediction results using different number of samples and general contribution (i.e. $PrISE_G$).

On the other hand, the best results using $PrISE_L$ are achieved using as few as 50 samples, as presented in Figure 5.

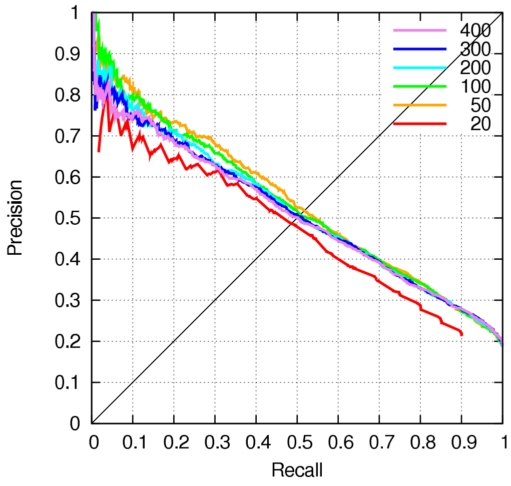

Figure 5: Prediction results using set of samples of different size and local contribution (i.e. $PrISE_L$).

The results of experiments using $PrISE_C$ presented in Figure 6 show that the prediction performances were similar when more than 300 samples were used. We decided to use 500 samples, which produced slightly better precision than the other alternatives for recall values between 0.6 and 0.75.

A comparison of the best results derived from all the previous experiments, as well as the curve computed from a randomized prediction, are shown in Figure 7. The randomized prediction was achieved by randomly shuffling the

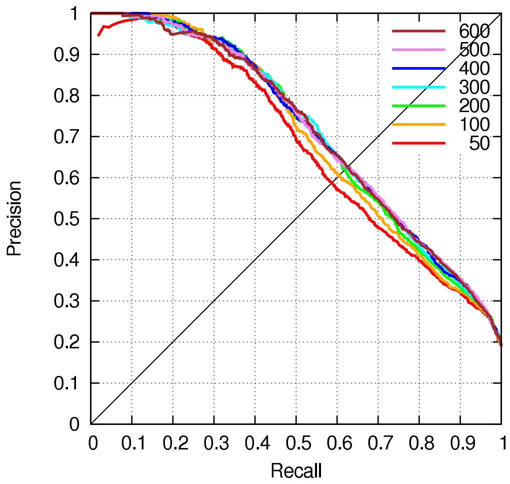

Figure 6: Prediction results using number of samples and combined contribution (i.e. $PrISE_C$).

interface/non-interface labels of the samples in the repository of structural elements, and performing prediction using samples weighted by combined contribution. From the figure it is possible to deduce that (i) all the prediction schemes are superior than random predictions, (ii) predictions generated using weighted samples are better than predictions produced using unweighted samples, (iii) schemes that incorporate general contribution produces better results than prediction based only in local contribution, and (iv) the best performance is achieved using the contribution scheme that combines local and general information.

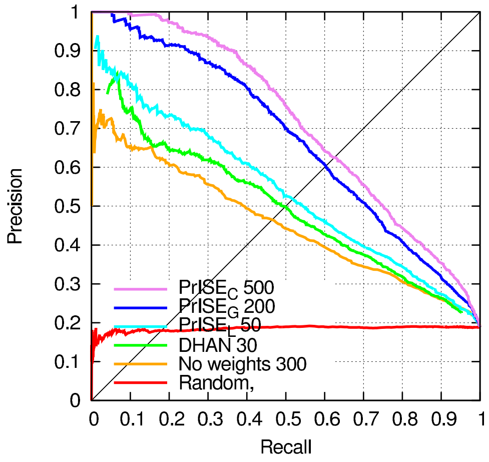

Figure 7: Prediction results using different weighting schemes. The number in the labels indicates the number of samples used for prediction.

As a result, the experiments described in the paper were performed using the top samples based on the city block

metric for DHAN, differences $\leq 5\%$ between the accessible surface areas of the central residues of the samples and the query structural elements, and differences $\leq 15\%$ between the accessible surface areas of the samples and each query structural element. The number of samples used by $PrISE_G$, $PrISE_L$, and $PrISE_C$ were set to 200, 50, and 500 respectively.

3.4 Selection of a threshold value for performing classification

The $PrISE$ family of predictors produce a probability that indicates the likelihood of each residue on the surface of the protein of being an interface residue. The selection of a threshold value on this probability allows to label each residue as interface / non-interface. The lower the threshold value, the more residues are labeled as interfaces. We used the results of the $PrISE_C$ predictor presented in Figure 7 to select a threshold value of 0.34, which produced predictions with similar precision and recall values. This value was used as default for all the predictors of the $PrISE$ family throughout the experiments presented in the paper .

4 Additional comparisons of $PrISE_L$, $PrISE_G$ and $PrISE_C$.

The performances of $PrISE_L$, $PrISE_G$ and $PrISE_C$ on the DS24Carl, DS56bound and DS56unbound datasets are shown in Figures 8 to 10. Samples extracted from homologs of the same species than the query proteins were filtered out from the repository of structural elements. In terms of performance, the precision recall curves indicate that $random < PrISE_L < PrISE_G \leq PrISE_C$.

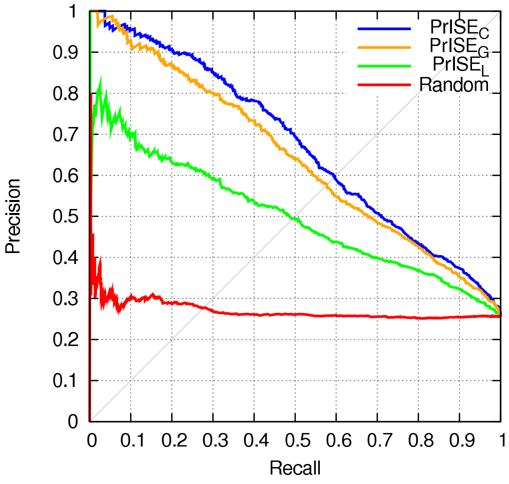

Figure 8: Comparison of $PrISE_L$, $PrISE_G$, and $PrISE_C$ using the dataset DS24Carl.

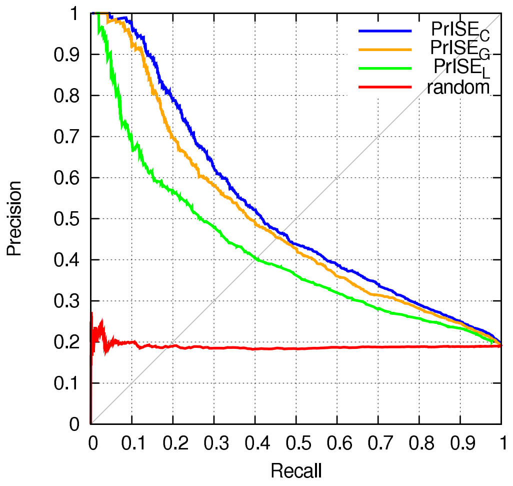

Figure 9: Comparison of $PrISE_L$, $PrISE_G$, and $PrISE_C$ using the dataset DS56Bound.

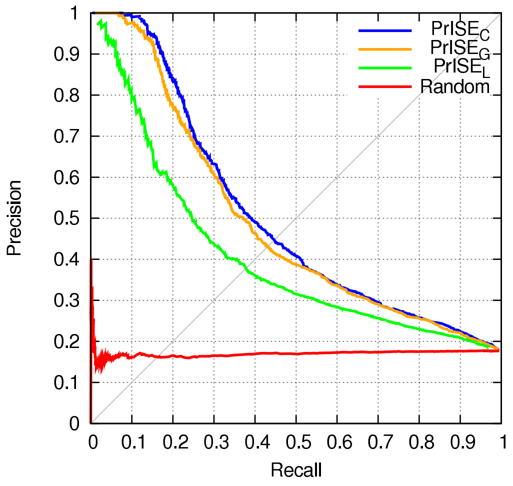

Figure 10: Comparison of $PrISE_L$, $PrISE_G$, and $PrISE_C$ using the dataset DS56Unbound.

An example of the relationship between the scores of $PrISE_L$, $PrISE_G$ and $PrISE_C$, and the actual interface/non-interface labels for some residues in the protein 1ohz-B is illustrated in Figure 11. From this figure is clear that $PrISE_C$ is successful correcting some erroneous predictions generated by both $PrISE_L$ and $PrISE_G$ (e.g. residues 19, 25, and 26) or by only one of them (e.g. amino acids 2, 18, and 24). $PrISE_C$ sometimes generates wrong predictions in cases where $PrISE_L$or $PrISE_G$ make correct predictions (e.g. residues 6, 11, 14, and 20). However, our experimental results indicate that the number of errors fixed by $PrISE_C$ exceeds the number of errors it introduces.

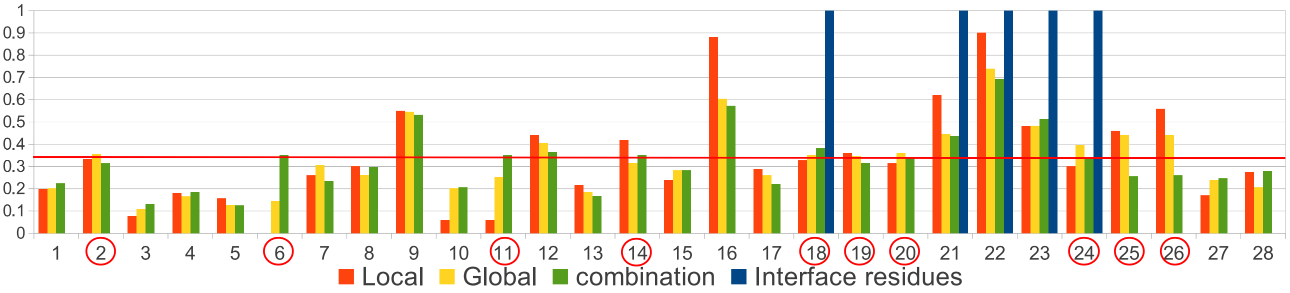

Figure 11: Example of the scores generated by $PrISE_L$,$PrISE_G$, and $PrISE_C$. This figure show (in the vertical axis) the score generated by $PrISE_L$,$PrISE_G$, and $PrISE_C$ as well as the actual interface residues for the first 28 residues (shown in the horizontal axis) in the sequence of the protein chain 1ohz-B. The horizontal red line signals the threshold computed on the scores (0.34) to differentiate between interfaces and non-interfaces.

5 Additional evaluation of the impact of homologs of the query protein in the predictions.

The impact caused on the predictions by filtering out from the repository samples derived from sequence homologs of the query proteins is presented in Figures 12 to 14. This evaluation was performed using $PrISE_C$ on the DS24Carl, DS56Bound and DS56Unbound datasets. These figures show that the prediction performances are lower when samples extracted from homologs of the query proteins are filtered out from the repository of structural elements.

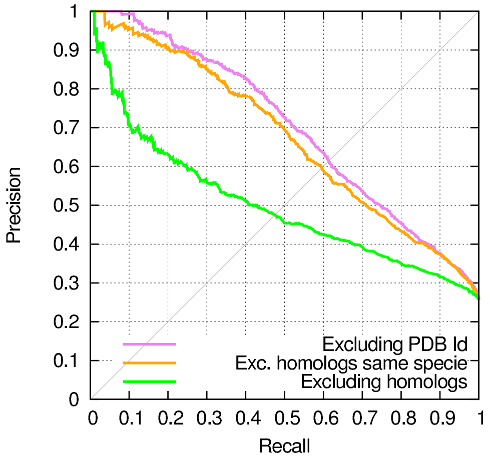

Figure 12: Performance of $PrISE_C$ with DS24Carl using three schemes for excluding similar proteins.

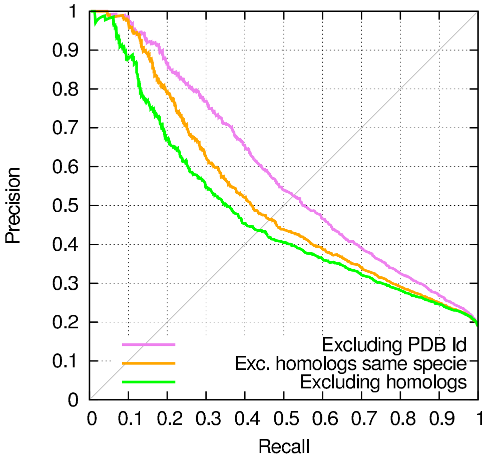

Figure 13: Performance of $PrISE_C$ with DS56Bound using different schemes for excluding similar proteins.

6 Additional comparison with two prediction methods based on geometrical conserved local surfaces.

A comparison of the predictors of the $PrISE$ family with the methods presented in [1, 2] using the DS24Carl dataset and excluding from the repository of structural elements samples extracted from homologs (without regarding the species) is presented in Table 3. According to this table, all the members of the $PrISE$ family outperform the classifiers presented in [1, 2] in terms of precision, recall, and F1.

We also evaluated the performance of the $PrISE$ family of predictors using the ProtInDb and the $ProtInDb \bigcap PQS$ repositories of structural elements. The results of these comparisons, shown in Tables 4 and 5, indicate that predictors that use samples extracted from the ProtInDb repository slightly outperform predictors that extract samples from the $ProtInDb \bigcap PQS$ respository.

Predictor	Precision %	Recall %	F1 %	Accuracy %	CC %	AUC %
Carl08	31.5	35.3	33.3	-	-	-
Carl10	32.0	34.0	33.0	-	-	-
$PrISE_L$	41.1	52.3	44.1	66.3	21.1	66.7
$PrISE_G$	45.6	48.6	45.4	69.9	24.0	68.8
$PrISE_C$	48.7	46.4	45.8	72.2	26.3	69.2

Table 3: Performance of different methods on the DS24Carl dataset. Performance measures are computed as the average on the set of 24 proteins. Precision and recall values for Carl08 and Carl10 were taken from [1] and [2] respectively. Samples derived from homologs of the query proteins were excluded from the ProtInDb repository.

Predictor	ProtInDb	Precision %	Recall %	F1 %	Accuracy %	CC %	AUC %
$PrISE_L$	✓	41.1	52.3	44.1	66.3	21.1	66.7
		41.0	50.7	43.3	66.6	19.2	66.6
$PrISE_G$	✓	45.6	48.6	45.4	69.9	24.0	68.8
		43.4	47.7	43.8	69.3	21.2	67.3
$PrISE_C$	✓	48.7	46.4	45.8	72.2	26.3	69.2
		45.5	47.7	45.0	70.4	23.4	69.5

Table 4: Performance of $PrISE$ predictors using different repositories of structural elements and excluding homologs. Performance measures are computed as the average on the set of 24 proteins in the DS24Carl dataset. Samples extracted from homologs (without regarding the species) were excluded from the prediction process. The column "ProtInDb" indicates whether samples were extracted from the ProtInDb repository (marked with a tick), or from the $ProtInDb \bigcap PQS$ repository.

Predictor	ProtinDb	Precision %	Recall %	F1 %	Accuracy %	CC %	AUC %
$PrISE_L$	✓	45.1	56.2	50.0	69.1	27.1	70.5
		46.3	55.7	48.6	69.9	26.8	71.0
$PrISE_G$	✓	53.9	58.7	56.2	75.1	36.8	75.6
		51.6	56.7	52.5	74.0	33.1	74.3
$PrISE_C$	✓	58.3	58.3	58.3	77.5	40.6	77.1
		54.4	58.4	54.8	75.5	36.6	76.2

Table 5: Performance of $PrISE$ methods using different repositories and excluding homologs of the same species. The performance measures were computed as the averages on the proteins in the DS24Carl dataset. Samples extracted from homologs from the same species than the query proteins were filtered out from the prediction process. The "ProtInDb" column indicates whether the samples were extracted from the ProtInDb repository (marked with a tick), or from the $ProtInDb \bigcap PQS$ repository.

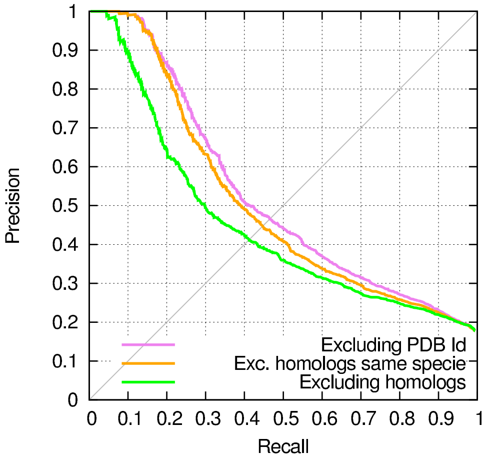

Figure 14: Performance of $PrISE_C$ with DS56Unbound using several schemes for excluding similar proteins.

7 Abbreviations

$dASAres$ - Difference between the accessible surface area of the central residues and of two structural elements.
$dASAse$ - Difference between the accessible surface area of two structural elements.
DHAN - distance between two histograms of atom nomenclatures.
Sample - a structural element retrieved from a repository of structural elements.

References

[1] N. Carl, J. Konc, and Dušanka Janežič. Protein surface conservation in binding sites. *J. Chem. Inf. Model*, 48(6):1279–1286, 2008.

[2] Nejc Carl, Janez Konc, Blaž Vehar, and Dusšanka Janežič. Protein-protein binding site prediction by local structural alignment. *J Chem Inf Model*, 50 (10), Oct 2010.

[3] S.H. Cha. Comprehensive survey on distance/similarity measures between probability density functions. *International Journal of mathematical models and methods in applied sciences*, 1(4):300–307, 2007.
